# Jacaranone Induces Apoptosis in Melanoma Cells via ROS-Mediated Downregulation of Akt and p38 MAPK Activation and Displays Antitumor Activity In Vivo

**DOI:** 10.1371/journal.pone.0038698

**Published:** 2012-06-06

**Authors:** Mariana H. Massaoka, Alisson L. Matsuo, Carlos R. Figueiredo, Camyla F. Farias, Natália Girola, Denise C. Arruda, Jorge A. B. Scutti, Paulete Romoff, Oriana A. Favero, Marcelo J. P. Ferreira, João H. G. Lago, Luiz R. Travassos

**Affiliations:** 1 Unidade de Oncologia Experimental, Universidade Federal de São Paulo, São Paulo, São Paulo, Brazil; 2 Centro de Ciências e Humanidades e Centro de Ciências Biológicas e da Saúde, Universidade Presbiteriana Mackenzie, São Paulo, São Paulo, Brazil; 3 Instituto de Ciências Ambientais, Químicas e Farmacêuticas, Universidade Federal de São Paulo, Diadema, São Paulo, Brazil; Duke University Medical Center, United States of America

## Abstract

**Background:**

Malignant melanoma is a deadly type of metastatic skin cancer with increased incidence over the past 30 years. Despite the advanced knowledge on the biology, immunobiology and molecular genetics of melanoma, the alternatives of treatment are limited with poor prognosis. On clinical trials, natural products and among them redox-active quinones have been tested in the attempt to control the growth of cancer cells. Recently, we isolated jacaranone from *Pentacalia desiderabilis*, a benzoquinone derivative that showed a broad antitumor activity and protective anti-melanoma effect in a syngeneic model. The purified substance is active at micromolar concentrations, is not hemolytic, and is not toxic in naïve mice.

**Methodology/Principal Findings:**

The jacaranone antitumor activity was shown against several human cancer cell lines *in vitro*. Moreover, the induction of apoptosis in murine melanoma cells and jacaranone antitumor activity *in vivo*, in a melanoma experimental model, were also shown. Jacaranone renders antiproliferative and proapoptotic responses in tumor cells, by acting on Akt and p38 MAPK signaling pathways through generation of reactive oxygen species (ROS). The free radical scavenger N-acetyl-cysteine (NAC) was able to completely suppress cell death induced by jacaranone as it blocked Akt downregulation, p38 MAPK activation as well as upregulation of proapoptotic Bax. Notably, treatment of melanoma growing subcutaneously in mice with jacaranone significantly extended the mean survival times in a dose-dependent manner.

**Conclusions/Significance:**

The results provide evidence for the mechanisms of action of jacaranone and emphasize the potential use of this quinone for the treatment of melanoma.

## Introduction

Among the conventional antitumor cytotoxic chemotherapies, many compounds are derived from natural products [1], [2], and commonly used agents to treat human cancers belong to the quinone class of organic compounds [3]. Antitumor quinones (e.g. daunorubicin and doxorubicin) can undergo reversible enzymatic reduction and oxidation, and form oxygen reactive species (ROS). The role of ROS as secondary messengers in intracellular signaling cascades has been investigated extensively in the last two decades [4]. There is evidence that low levels of ROS can induce cell proliferation and genetic instability, which may contribute to the oncogenic phenotype of cancer cells. At high concentration ROS can promote cellular senescence and apoptosis, and therefore may function as antitumorigenic species [5]. Indeed, cancer cells frequently exhibit abnormal redox status associated with increased basal production of ROS, and thus cannot tolerate higher levels of free radicals [6]. Therefore, the use of compounds that interfere in redox regulation is a promising strategy to selectively target tumors [4].

Recently, we isolated jacaranone from leaves of *Pentacalia desiderabilis* [7]. Although potential antiproliferative protective effects have been described for extracts and purified jacaranone from *Senecio leucanthemifolius* Poiret [8], the mechanism of action and the antitumor activity of this quinone has not been previously investigated. In the present work, we show that jacaranone induces biochemical and morphological alterations of apoptosis mediated by generation of reactive oxygen species (ROS). Investigation of the signaling pathways affected by jacaranone showed downregulation of Akt, activation of p38 MAPK and upregulation of pro-apoptotic Bax. We also evidenced the antitumor potential of jacaranone as a therapeutic anticancer drug using several human cancer cells *in vitro*. In particular, we showed the protective effect of this quinone in a melanoma syngeneic model *in vivo*, using B16F10-Nex2 cells grafted subcutaneously in mice. The antitumor and protective effects of jacaranone are highly significant at low therapeutic doses.

## Materials and Methods

### Ethics statement

All necessary permits were obtained for the described field studies. The State of São Paulo Research Support Foundation (FAPESP), Brazil, and the Brazilian National Research Council (CNPq) provided permits to collect plant material. The site of plant collection is privately owned by Mackenzie Presbyterian University, where the authors carried out the plant collection work. We ensure that the procedures involving plant material were applied in accordance with label guideline and the field studies did not involve endangered or protected species. Cell lines were originally obtained from Prof. Luiz Fernando Lima Reis, Ludwig Institute for Cancer Research, São Paulo, Brazil. These are long established cell lines, acquired from public culture collections or transferred from Ludwig Institute in New York, and maintained in appropriate conditions to serve as standard tumor cell lines for local studies and collaborative research. We confirm that animal experiments were carried out using protocols approved by the Ethics Committee for Animal Experimentation of Federal University of São Paulo, Brazil and the specific Project presented by the Experimental Oncology Unit, including the animal experiments herein reported, has been approved via doc CEP 1234/2011.

### Plant material

Leaves from *Pentacalia desiderabilis* (Vell.) Cuatrec. were collected in Campos do Jordão, São Paulo, SP, in August 2008. A voucher specimen was deposited at Herbarium of D. Bento Pickel – Instituto Florestal under number SPSF 37596.

### General Procedures

^1^H (300 MHz) and ^13^C (75 MHz) NMR spectra were obtained on a Bruker (Billerica, MA) model DPX-300 spectrometer with sample dissolved in CDCl_3_ containing 1% of tetramethylsilane (TMS, TediaBrazil, Rio de Janeiro, Brazil). The LREIMS (low resolution electron impact mass spectrum) was obtained at 70 eV on Finnigan-Mat INCOS50 quadrupole spectrometer. Chromatographic separation procedures were performed using silica gel (230–400 mesh; Merck, Darmstadt, Germany) for CC and silica gel 60 PF_254_ (Merck, Darmstadt, Germany) for analytical TLC (0.25 mm).

### Isolation of jacaranone from leaves of *P. desiderabilis*

Dried and powdered leaves of *P. desiderabilis* (232 g) were defatted with hexane and exhaustively extracted with methanol. After solvent evaporation under reduced pressure, the crude methanol extract (13.8 g) was dissolved in methanol : H_2_O 1∶2 and partitioned sequentially using hexane and CH_2_Cl_2_. After solvent evaporation under reduced pressure, the following yields were obtained: 0.41 g /hexane and 2.50 g /CH_2_Cl_2_ phases. Part of CH_2_Cl_2_ phase (0.4 g) was subjected to silica gel column chromatography eluted with CH_2_Cl_2_ containing increasing amounts of ethyl acetate (up to 100%) and ethyl acetate containing increasing amount of methanol (up to 100%), to give twelve fractions (A1 – A12). Jacaranone was isolated as colourless needles (132.5 mg) from fraction A4. Jacaranone [Methyl (1-hydroxy-4-oxo-2,5-cyclohexandienyl) acetate]. Colourless needles; ^1^H NMR (CDCl_3_, 300 MHz): δ 6.97 (d, J = 10.2 Hz, H-2′/H-6′), 6.21 (d, J = 10.2 Hz, H-3′/H-5′), 3.75 (s, OCH_3_), 2.72 (s, H-2). ^13^C NMR (CDCl_3_, 75 MHz): 171.0 (C-1), 43.4 (C-2), 67.3 (C-1′), 149.0 (C-2′/C-6′), 128.2 (C-3′/C-5′), 185.0 (C-4′), 52.2 (OCH_3_). LREIMS m/z (rel. int.): 182 [M^+^] (3), 166 (2), 150 (19), 122 (16), 109 (84), 94 (8), 81 (44), 74 (100), 69 (7), 59 (19), 53 (36).

### Cell lines and culture conditions

Murine melanoma subline B16F10-Nex2 was established at the Experimental Oncology Unit (UNONEX), Federal University of São Paulo, UNIFESP, as described previously [9]. The human melanoma cell lines A2058 and SK-MEL-28, human colon carcinoma cell lines HCT-8 and LS160, human cervical carcinoma cell line SiHa, human myeloid leukemia cells HL-60, human breast cancer cells MDA and SK-BR-3, and the murine melanocytes melan-A cell line were provided by the Ludwig Institute for Cancer Research, São Paulo, Brazil. These cell lines were maintained in complete medium consisting in RPMI-1640 (Gibco, Grand Island, NY) supplemented with 10 mM N-2-hydroxyethylpiperazine-N2 ethanesulphonic acid (HEPES; Sigma-Aldrich, St. Louis, MO), 24 mM sodium bicarbonate, 40 mg/l gentamicin (Hipolabor, Minas Gerais, Brazil), pH 7.2, and 10% fetal bovine serum (Gibco, Grand Island, NY) at 37°C in a humidified atmosphere with 5% CO_2_.

### Preparation of murine bone marrow cells and macrophage differentiation

Fresh bone marrow cells were used to generate macrophages using L929-cell supernatant conditioned medium (LCCM) as a source of granulocyte/macrophage-colony stimulating factor (GM-CSF). The cells were resuspended in 10 ml of bone marrow differentiation medium, which consists of RPMI1640 (Gibco, Grand Island, NY) supplemented with 20% fetal bovine serum (Gibco, Grand Island, NY), 30% LCCM, 100 U/ml penicillin, 100 µg/ml streptomycin, and 2 mM L-glutamine. Cells were seeded in non-tissue culture treated Optilux Petri dishes (BD Biosciences, Franklin Lakes, NJ) and incubated at 37°C in a 5% CO_2_ atmosphere. After 4 days, 10 ml of fresh medium was added per plate and incubated for additional 3 days. To obtain the macrophages, the supernatants were discarded and the attached cells were washed with 15 ml of sterile PBS. Macrophages were detached gently using a cell scraper and PBS. The cells were centrifuged at 200 *g* for 5 minutes and resuspended in 10 ml of RPMI 1640 (Gibco, Grand Island, NY). The cells were counted, seeded and cultivated in tissue culture plates for 12 h.

### Cell viability assay

Viable cells were quantified using the MTT (3-[4,5-dimethylthiazol-2-yl]-2,5-diphenyltetrazolium bromide) (Sigma-Aldrich, St. Louis, MO) assay. Human tumor cell lines or B16F10-Nex2 (1.0×10^4^ cells/well) were cultured on 96-well plates in RPMI supplemented with 10% serum, and jacaranone (1.0, 2.5, 5.0, 10.0, 20.0 or 50.0 µM) or fresh medium (for control cells) was added 8 h after the tumor cell inoculum. After 24 h, MTT solution (5 mg/ml) in 1× phosphate-buffered saline (PBS) was directly added to the cells, followed by incubation for 4 h at 37°C. Absorbance was measured with an automated spectrophotometric plate reader (SpectraMax-M2, Molecular Devices Software Pro 5.4, Sunnyvale, CA) at a wavelength of 570 nm. Cell viability was expressed as percent values in comparison with untreated cells.

### Annexin V and propidium iodide labeling

B16F10-Nex2 cells (5×10^5^ cells/well) were cultured in 6-well plates and further incubated with 20 or 50 µM jacaranone or complete medium (control) for 24 h at 37°C. Treated and untreated cells (1.0×10^6^) were washed three times with PBS and harvested with a cell scraper. Apoptotic/necrotic cells were detected using the Annexin V-FITC Apoptosis Detection Kit (Sigma-Aldrich, St. Louis, MO). Cells were incubated with binding buffer (10 mM HEPES/NaOH, pH 7.5, 140 mM NaCl and 2.5 mM CaCl_2_) in presence of propidium iodide (PI) and FITC-labeled annexin V (AV) for 10 min at room temperature and analyzed by flow cytometry (BD Bioscience FACSCanto II equipment, Franklin Lakes, NJ), using FlowJo software (TreeStar Inc., Ashland, OR).

### DNA degradation

B16F10-Nex2 cells (5×10^5^/well) cells were seeded on 6-well plates and incubated for 12 h at 37°C. Cells were then treated with 100 µM jacaranone or left untreated, and incubated at 37°C for 18 h. DNA was isolated after cells were lysed in TELT buffer (50 mM Tris–HCl pH 8.0, Triton X-100 0.4%, 2.5 mM EDTA pH 9.0, and 2.5 M LiCl). The lysate was centrifuged for 20 min (12,000 *g*) at 4°C. Buffer-equilibrated phenol was added (1∶1, v/v), followed by centrifugation and addition of chloroform (1∶1, v/v) to the resulting aqueous phase. Tubes were centrifuged (15 min, 12,000 *g*, 4°C), the aqueous phase was collected and DNA was precipitated with sodium acetate 3 M, pH 7.0 and absolute ethanol (1∶0.1∶2.5, v/v/v) after incubation at – 80°C for 20 min. Precipitated DNA was pelleted and diluted in 50 µg/ml of RNAse-A (Invitrogen, Carlsbad, CA). The extracted DNA was subjected to electrophoresis on 1% Agarose gel with ethidium bromide (0.5 μg/ml) in TBE buffer (2 mM EDTA, 90 mMT ris–HCl, 90 mM boric acid, pH 8.0) at 100 V. One thousand base-pair (1 kb) ladder molecular weight markers (Gibco, Grand Island, NY) were used. After electrophoresis, DNA was photographed with a digital camera (Kodak, EDAS DC290) under ultraviolet (UV) illumination.

### TUNEL Assay

Apoptotic cell death was also assessed by terminal deoxynucleotidyltransferase dUTP nick end-labeling (TUNEL) assay kit (In Situ Cell Death Detection Kit, Fluorescein, Roche Applied Science, Indianopolis, IN), according to manufacturer's instructions. Briefly, 1.0×10^4^ B16F10-Nex2 cells were seeded onto round glass coverslips and cultivated for 24 h before incubation with 50 µM jacaranone for 3 h. After treatment, cells were washed with PBS and fixed with 2% formaldehyde for 30 minutes at room temperature. Next, cells were permeabilized with 0.1% Triton X-100 for 30 min at room temperature and incubated with TdT enzyme (terminal deoxynucleotidyltransferase) in the reaction buffer with dUTP-fluorescein at 37°C for 1 h, followed by staining with 10 µg/mL of 4′,6-diamidino-2-phenylindole (DAPI, Invitrogen, Carlsbad, CA) for 10 min in the dark. Apoptotic cells were visualized on Olympus BX-51 fluorescence microscope using 60X oil immersion objective. Images were processed with ImageJ (http://rsb.info.nih.gov/ij/). As a positive control, cells were treated with 1 mg/mL actinomycin D (Sigma-Aldrich, St. Louis, MO) for 1 h.

### Detection of caspase activity by colorimetric assay

For evaluation of caspase activity, B16F10-Nex2 cells (1.0×10^6^) were cultured and treated with or without 20 µM jacaranone. After the indicated periods, cells were harvested and tested for caspase activity using the ApoTarget Caspase Colorimetric Protease Assay Sampler Kit (Invitrogen, Carlsbad, CA) according to manufacturer's protocol. Briefly, the cells were resuspended in 50 μL of chilled Cell Lysis Buffer for 10 min on ice bath. The lysates were centrifuged at 10,000 *g* for 1 min, and 200 µg of the protein, determined by Bradford’s method [10] was incubated with 200 μM substrate VDVAD-*p*NA (caspase-2), DEVD-*p*NA (caspase-3), IETD-*p*NA (caspase-8), and LEHD-*p*NA (caspase-9) at 37°C for 2 h in a 96-well plate. Absorbance of *p*NA was read at 405 nm in a microplate reader (SpectraMax-M2, Software Pro5.4, Molecular Devices, Sunnyvale, CA).

### Chromatin condensation analysis

Murine melanoma cells (1.0×10^4^) were cultivated overnight on round glass coverslips and incubated with or without 20 or 50 µM jacaranone for 3 h. Then, cells were washed with PBS, fixed for 30 min at room temperature with 2% formaldehyde, and stained with 2 µM Hoechst 33342 (Invitrogen, Carlsbad, CA) for 10 min to study the state of chromatin. Cells were visualized on an Olympus BX-51 fluorescence microscope with immersion oil at 60X magnification. Apoptotic cells were detected by condensed or fragmented nuclei.

### Apoptotic morphological changes by transmission electron microscopy

Transmission electron microscopy (TEM) was used to provide ultrastructural evidence of jacaranone-induced apoptosis. B16F10-Nex2 cells (5×10^4^) were seeded on 12-mm round aclar plastic coverslips housed in 6-well plates, and incubated with 50 µM jacaranone for 3 h at 37°C. After fixation with 2.5% glutaraldehyde and 2% formaldehyde in 0.1 M sodium cacodylate buffer, pH 7.2 at room temperature for 20 h, cells were washed in the same buffer for 10 min, and then post-fixed with 1% osmium tetroxide in 0.1 M cacodylate at pH 7.2 for 30 minutes. Next, cells were washed with distilled water for 10 minutes at room temperature, treated with an aqueous solution of 0.4% uranyl acetate for 30 minutes and washed again with distilled water for 10 minutes. After fixation, cells were dehydrated in a graded series of ethanol (70%, 90% and 100%) and propylene oxide washes, and embedded in Epon. Semi-thin sections were obtained from selected areas, collected on copper grids, stained with 1% uranyl acetate and lead citrate, and examined on a Jeol 1200 EXII electron microscope (Tokyo, Japan).

### Detection of mitochondrial membrane potential (Δ*ψ*m)

The cationic lipophilic dye tetramethylrhodamine ethyl ester (TMRE) enters the cell in the form of an ester that is subsequently hydrolyzed and converted to tetramethylrhodamine, which is reversibly accumulated in the negatively charged mitochondrial matrix depending on mitochondrial membrane potential. To analyze the Δ*ψ*m after jacaranone treatment, 1.0×10^5^ B16F10-Nex2 cells were grown for 24 h in a 12-well culture plate and incubated with 50 µM jacaranone for the indicated times at 37°C. Cells were gently washed with PBS and loaded with 20 nM TMRE (Molecular Probes, OR, USA) for 10 min at 37°C. Cells were detached with PBS/Trypsin/EDTA and the ﬂuorescence was measured on FACSCanto II (BD Bioscience, Franklin Lakes, NJ), using FACSDiva software (BD Bioscience, Franklin, NJ) and analyzed using FlowJo software (TreeStar Inc., Ashland, OR, USA).

### Measurement of intracellular superoxide anion

Superoxide anion production was evaluated by measuring changes in fluorescence resulting from intracellular probe oxidation. Dihydroethidium (DHE) assay was performed according to manufacturer's instructions (Invitrogen, Carlsbad, CA). Briefly, 1.0×10^4^ cells were cultivated on round glass coverslips and treated with 10 µM jacaranone for 3 h at 37°C. Analysis by fluorescence microscopy was carried out on Olympus BX-51 microscope using 60X oil immersion objective. Images were processed with ImageJ (http://rsb.info.nih.gov/ij/). As a positive control, cells were treated with 5 mM H_2_O_2_ at 37°C for 20 minutes.

### Determination of N-acetylcysteine (NAC) effects on jacaranone-induced cell death

Murine melanoma B16F10-Nex2 cells (5×10^3^/well) were cultured in 96-well plates in the presence or absence of NAC at 1, 2 or 4 mM for 30 min. Subsequently, 20 µM jacaranone was added and incubated for 18 h at 37°C. After the incubation period, cell viability was obtained by Trypan Blue (Gibco, Grand Island, NY) dye exclusion test.

### Redox cycling induced by jacaranone

To evaluate the jacaranone-catalyzed redox cycling ability, the nitroblue tetrazolium (NBT, Sigma Aldrich, St. Louis, MO)/glycinate assay was performed as described previously [11]. NBT/glycinate assay is a colorimetric test based on the fact that quinones, at an alkaline pH, can oxidize glycine in an oxidative amine degradation reaction. The hydroquinones or semiquinones formed react with molecular oxygen, which is monitored by the reduction of NBT to formazan [12]. NBT/glycinate assay was performed in 96-well microtiter plates, using 60 µl jacaranone sample at different final concentrations of 20, 10, 5, 2.5, 1.5 or 0.65 µM ; 20 µl BSA (initial concentration of 50 mg/ml in 10 mM potassium phosphate, pH 7), and 200 µl NBT/ glycinate reagent (0.24 mM NBT in 2 M potassium glycinate, pH 10). After incubation at 37°C for 1 h, the absorbance was read at 605 nm.

### Cell lysate extracts and Western blotting

For protein extraction, 1.0×10^6^ B16F10-Nex2 cells treated with 20 or 50 µM jacaranone for 24 h and untreated cells (control) were washed with PBS and lysed by adding 100 µL of 1x SDS sample buffer (62.5 mM Tris-HCl, pH 6.8 at 25°C, 2% w/v SDS, 10% glycerol, 50 mM DTT, 0.01% w/v bromophenol blue). Lysates were transferred to microcentrifuge tubes, and kept on ice. After sonication for 15 seconds, samples were heated to 95°C for 5 min and centrifuged at 1,500 rpm for 10 min. Total proteins from each cell lysate were separated in SDS gel electrophoresis and Western blotting was carried out as described elsewhere [13]. The following primary antibodies were used: rabbit anti-β-actin, rabbit anti-phospho-p38 (Thr180/Tyr182) or total-p38, rabbit anti-phospho Akt (Thr308) or total-Akt, all purchased from Cell Signaling Technology (Beverly, MA), and mouse anti-Bax (Merck, Darmstadt, Germany). β-actin was used as loading control. Secondary antibodies conjugated with IgG horseradish peroxidase were purchased from Sigma-Aldrich (St. Louis, MO) and immunoreactivity was detected using the Immobilon solution (Millipore, Billerica, MA). To evaluate the involvement of ROS in jacaranone-mediated effects, lysates from cells preincubated with 4 mM NAC and exposed to 20 or 50 µM jacaranone were obtained and subjected to immunoblottings as described above.

### Animals and subcutaneous tumor growth

Eight-week-old male C57BL/6 mice were obtained from the Center for Development of Experimental Models (CEDEME), Federal University of São Paulo (UNIFESP). Animal studies were conducted in compliance with UNIFESP Ethics Committee for Animal Experimentation.

The therapeutic efficacy of jacaranone was examined in syngeneic B16F10-Nex2 melanoma-bearing mice. C57BL/6 mice were inoculated subcutaneously (s.c.) with 5×10^4^ viable melanoma cells in the right flank. Two treatment groups of 5 mice were subjected to seven intraperitoneal (i.p.) doses of 0.8 mg/kg or 4 mg/kg of jacaranone (diluted in PBS). An untreated tumor control group received 100 µL of PBS. Treatment started one day after tumor cell implantation and was administered on alternate days in a period of 14 days. After administration of the therapeutic doses, the tumor size was measured daily with a caliper. Tumor volume (V) was calculated by the formula V = 1/2×d^2^×D, where d and D are the short and long diameters of the tumor, respectively. Mice were removed from the therapy study and sacrificed when tumor size exceeded 3,000 mm^3^.

The protection experiment was repeated for confirmation with a group of 6 animals, using 4 mg/kg of jacaranone and doxorubicin (0.1 mg/kg, Sigma-Aldrich, St.Louis, MO) as a positive control.

### Statistical analysis

Unless otherwise specified, two independent experiments were performed in triplicate. Experimental values are expressed as mean value ± standard deviation (S.D.). For significance analyses Student's t-tests were calculated using GraphPad Prism 4.0 software (La Jolla, CA). *p* values <0.05 were considered significant.

## Results

### Cytotoxic effects of jacaranone on cancer cells

**Figure 1:**
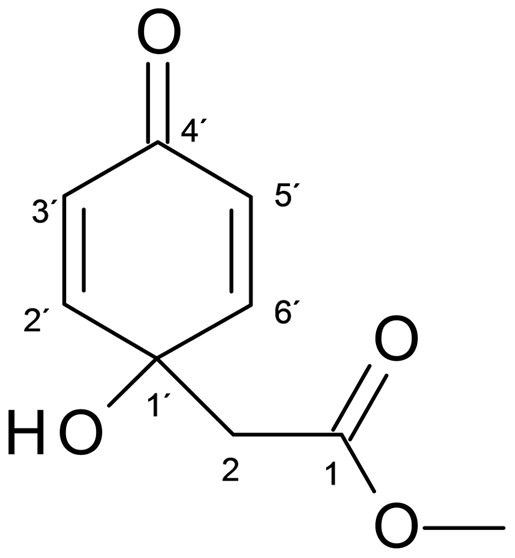
Chemical structure of jacaranone isolated from leaves of *P.* *desiderabilis* *.*

The cytotoxicity of jacaranone [Methyl (1-hydroxy-4-oxo-2,5-cyclohexandienyl) acetate] (Fig. 1) was determined on human cancer cell lines and the murine melanoma B16F10-Nex2 cell line. In all tested cell lines jacaranone induced cell death *in vitro* in a dose-dependent manner and showed different degrees of cytotoxicity. As observed in Table 1, IC_50_ values (the concentration of jacaranone that reduced cell survival by 50%) varied from 9 to 145 µM for human cancer cells. The IC_50_ for murine melanoma B16F10-Nex2 was 17 µM.

**Table 1:** Cytotoxic activity of jacaranone on human cancer cells growing in vitro.

Cell line	IC_50_ (µM)^a^
A2058	30
SK-MEL-28	23
B16F10-Nex2	17
HCT-8	24
LS160	36
SiHa	145
HL-60	9
SK-BR-3	30
MDA	77

a IC_50_ is the concentration that kills 50% of viable cells in a dose-dependent survival curve.

### Jacaranone induces apoptosis in melanoma cells

**Figure 2:**
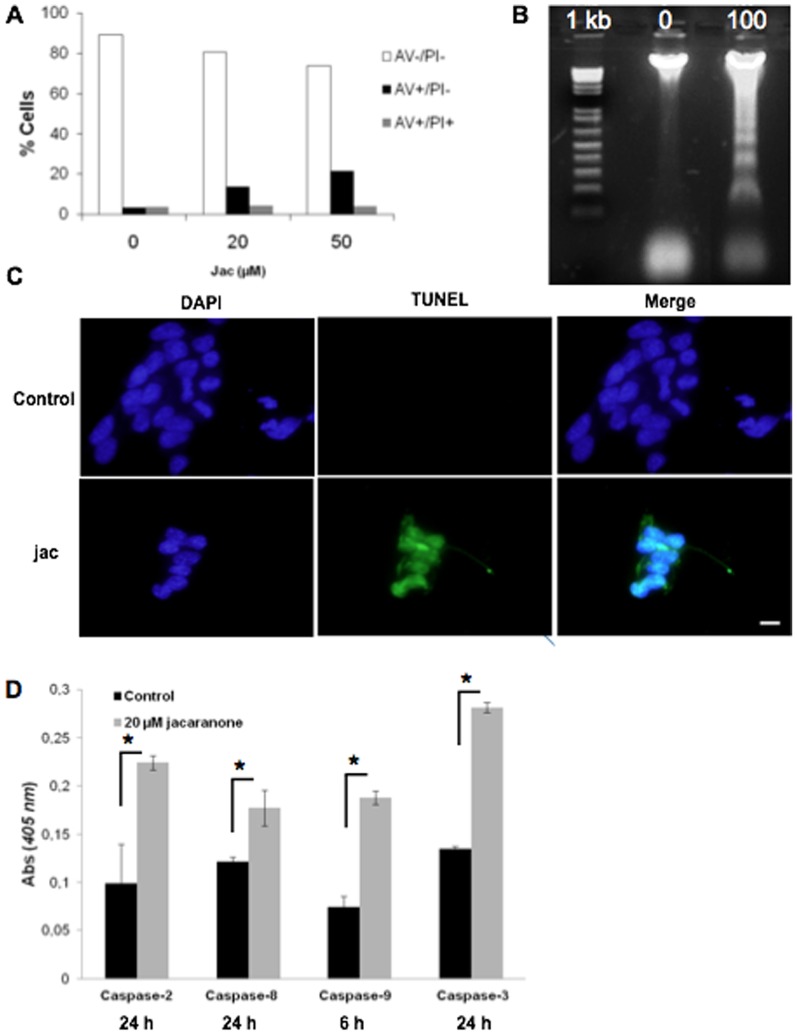
Jacaranone suppressed cell proliferation and induced apoptosis in melanoma cells. (A) 5×10^5^ B16F10-Nex 2 cells were seeded onto 6-well plates and treated with 20 or 50 µM jacaranone at indicated times. The cells were incubated with a FITC-conjugated annexin V and PI solution, and then analyzed by flow cytometry. (B) B16F10-Nex2 cells were incubated with or without 100 µM jacaranone for 18 hours. The isolated DNA was electrophoresed and fragments were visualized by staining with ethidium bromide under UV light. (C) After treatment with 50 µM jacaranone (Jac) for 3 h, the cells were fixed and incubated using TUNEL reaction solution and then visualized under a fluorescence microscope as described in experimental procedures. (D) Cells were treated with or without jacaranone at 20 µM for different times, harvested, and cell lysates were tested for caspase-2, -8, -9 and -3 activities. Results are representative of at least two independent experiments, and shown as mean ± S.D. *p ≤0.02 versus control conditions.

To evaluate the cytotoxic activity, we investigated whether cell death induced by jacaranone was associated with biochemical features of apoptosis [14]. Treatment of melanoma cells with jacaranone elicited a concentration-dependent phosphatidyl serine (PS) translocation (Fig. 2A) as determined by annexin-V assay. The early apoptotic cells (regarded as annexin V positive and PI negative cells) increased as compared with the non-treated cells. A very low percentage of annexin-V positive cells (3.45%) was observed in the non-treated population. Jacaranone at 20 and 50 µM induced apoptosis in 13.8 and 21.5% of cells, respectively. In this experiment, both concentrations were sublethal due to the high number of cells. On looking for DNA fragmentation, a ladder pattern of internucleosomal fragmentation was observed after treatment with jacaranone at 100 µM for 18 h (Fig. 2B). In addition, DNA strand breaks of apoptotic cell death were visualized by TUNEL assay under fluorescence microscope (Fig. 2C). Since caspases play a major role in the execution of apoptosis, we therefore assessed the activity of initiator caspases -2, -8 and -9 and effector caspase-3. After treatment of B16F10-Nex2 cells for different times (2 h, 3 h, 6 h, 12 h and 24 h), caspase activation was assessed. The levels of caspase-2, -8, and -3 activities were 2-fold, 1.3-fold, and 2-fold higher, respectively, in 20 µM jacaranone-treated cells for 24 h compared with the control, all of which were significant (*p <0.05) (Fig. 2D). Activation of caspase-9 occurred 6 h after treatment and its activity was 2.5-fold higher when compared to untreated cells.

### Melanoma cells treated with jacaranone exhibit morphological alterations of apoptosis

**Figure 3:**
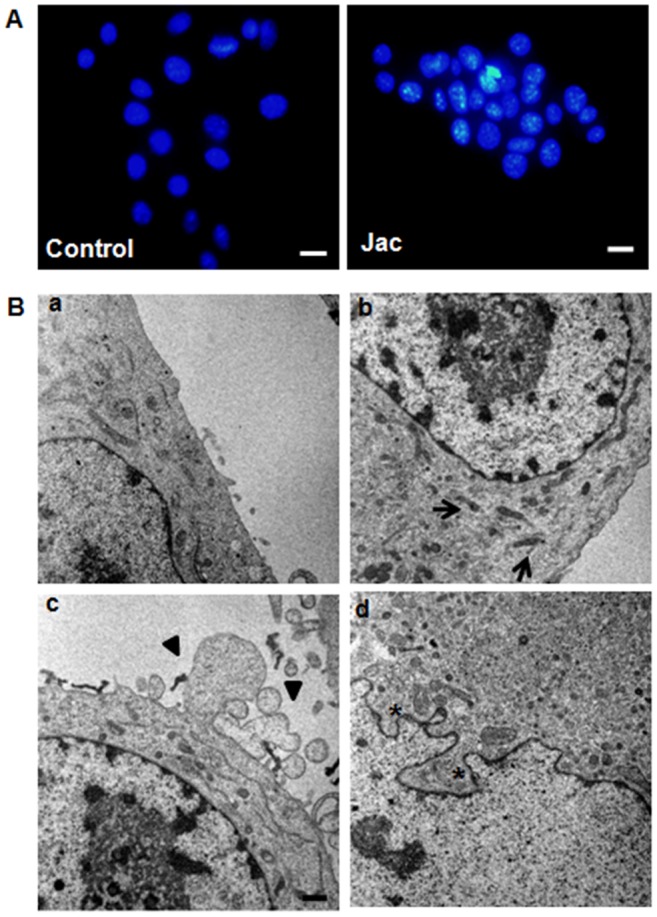
Jacaranone induced apoptotic morphological alterations on melanoma cells. (A) 1.0×10^4^ B16F10-Nex2 cells were seeded on coverslips and treated with 20 or 50 µM jacaranone (Jac) for 3 h. State of chromatin was assessed using Hoechst 33342 (2 µM). Positive cells for Hoechst staining were visualized under a fluorescence microscope and expressed as percentage of total cell counts of control cells. Representative images of cells treated without (Control) or with 20 µM jacaranone. (B) Transmission electron micrographs revealed mitochondrial shrinkage (arrows), plasma membrane blebbing (black triangles) and disruption of nuclear membrane (asterisk) after treatment with (b, c, and d) or without (a) 50 µM jacaranone. Original magnification ×10,000 (a, b and c); bar = 2 µm. Original magnification ×20,000 (d); bar = 1µm.

In order to study the details of apoptotic morphological changes [14] displayed by jacaranone in B16F10-Nex2 cells, Hoechst staining and transmission electron microscopy were used. The density of nuclear chromatin was analyzed by culturing cells on cover slips in the presence of 20 or 50 µM jacaranone for 3 h. Hoechst 33342 dye was added and its fluorescence was captured in the 4,6-diamidino-2-phenylindole channel. Fluorescence observation confirmed that cells treated with jacaranone exhibited condensation as in apoptotic nuclei (Fig. 3A). The percentage of apoptotic nuclei was 25.5% and 39.5% in melanoma cells incubated with 20 and 50 µM jacaranone, respectively. For TEM, approximately 5×10^4^ cells were incubated with 50 µM jacaranone for 3 h. Then, sections were made, as described in experimental procedures. Micrographs showed that jacaranone caused mitochondrial shrinkage (arrows), blebbing of plasma membrane (asterisks), and nuclear membrane disruption (black triangles) (Fig. 3B). These results, collectively with biochemical features [14], [15] demonstrated on Fig. 3, confirmed that jacaranone-treated cells undergo apoptosis.

### Jacaranone induces programmed cell death through oxidative stress and causes mitochondrial depolarization

**Figure 4:**
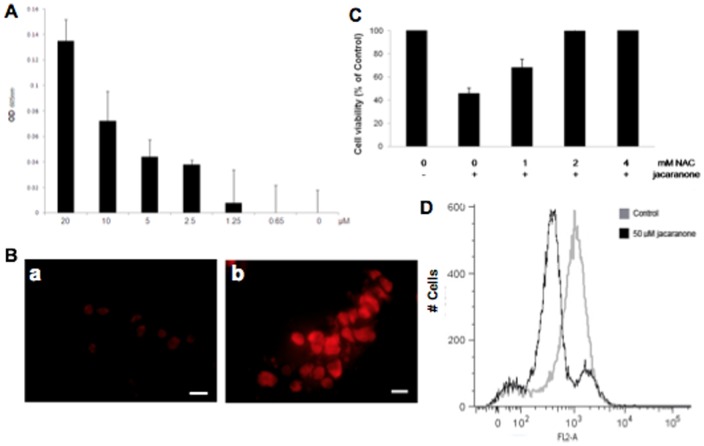
ROS generation by jacaranone and loss of mitochondrial transmembrane potential (Δ*ψ* **m) in jacaranone-treated cells.** (A) Redox cycling ability of jacaranone detected by NBT/glycinate assay. (B) Untreated melanoma cells (a) or cells treated with jacaranone at 50 µM for 3 h (b), were incubated with the oxidative fluorescent dye DHE (5 µM) for detection of superoxide anion production. Bars = 20 µm. (C) B16F10-Nex2 cells were pre-incubated with NAC at indicated concentrations for 30 min, and then jacaranone at 20 µM was added. Cell viability was assessed using Trypan Blue exclusion test 24 h after jacaranone addition. (D) B16F10-Nex2 cells were treated with 50 µM jacaranone for 24 h and then Δ*ψ*m was determined using TMRE by flow cytometry.

Previously, it was found that jacaranone exhibits weak scavenging effect on 1,1-diphenyl-2-picryl- hydrazyl (DPPH) radical [16]. To better characterize the molecule, we further investigated whether jacaranone exhibited redox-cycling ability, a well-known chemical property of many quinoid compounds. Using the NBT/glycinate assay we demonstrated for the first time that jacaranone is markedly efficient in redox cycling (Fig. 4A). The cycling reaction of jacaranone reduces nitroblue tetrazolium to formazan, resulting in increased optical density measured at 605 nm.

It has been reported that increased liberation of reactive oxygen species (ROS) can trigger a cascade of events leading to apoptosis [17]. Moreover, previous studies demonstrated that quinones induce tumor cells death through redox-mediated mechanisms [18], [19]. We have further investigated whether the production of intracellular ROS, as induced by jacaranone, could mediate apoptosis in melanoma cells (Fig. 4B). After 3 h treatment with 50 µM jacaranone, cells were stained with dihydroethidium (DHE) for 30 minutes at 37°C and its conversion to ethidium by oxidation was assessed under a fluorescence microscope. As observed, treatment with jacaranone resulted in highly increased ethidium fluorescence intensity as compared to non-treated cells. In this experiment, the jacaranone concentration was sublethal by itself due to the high number of cells.

Growing evidence suggests that cancer cells, relative to normal cells, have increased generation of ROS and an altered redox status [20]. To determine whether oxidative stress mediated by jacaranone contributed to the differential susceptibility of melanoma cells to ROS generation in comparison with normal cells, the oxidation of DHE was also measured in murine melanoma cells (B16F10-Nex2), murine melanocytes (melan-A) and primary culture of bone marrow macrophages from C57BL/6 mice after treatment with 50 µM jacaranone for 3 h. In melanoma cells ROS production increased by 50–57%, whereas in normal murine melanocytes and primary macrophages it increased only 18–19.5% and 4–5% respectively (data not shown).

When B16F10-Nex2 cells were pre-incubated with N-acetyl cysteine (NAC), a ROS scavenger, at 2 or 4 mM for 30 min before jacaranone treatment, cell death was completely abrogated (Fig. 4C). A nuclear magnetic resonance analysis excluded any direct interaction between jacaranone and NAC (data not shown).

The involvement of caspases-2 and -9 in the jacaranone-induced apoptotic death of melanoma cells and the increased ROS production suggested that jacaranone treatment might act by disrupting the mitochondrial membrane. We therefore performed a time-course analysis of the mitochondrial transmembrane potential using the lipophilic cationic dye TMRE to evaluate the effect of jacaranone on melanoma cells. A 0.5–1 h exposure to 50 μM jacaranone was found to induce an initial hyperpolarization of the mitochondrial membrane reflected by increase in fluorescence intensity as compared to untreated cells (data not shown). However, after 2 and 4 h of treatment, fluorescence intensity decreased significantly, suggesting that jacaranone treatment resulted in dissipation of Δ*Ψ*m. After 24 h of treatment, the mitochondrial damage during jacaranone-induced apoptosis in B16F10-Nex2 cells was shown by reduction in 51% of the accumulation of TMRE in the organelle (Fig. 4D) thus implying a decreased permeability threshold.

Taken together, these data strongly suggest that jacaranone produces ROS through its redox cycling capacity, and ROS mediates jacaranone-induced apoptosis in melanoma cells.

### Downregulation of Akt, activation of p38 MAPK and induction of Bax expression by jacaranone

**Figure 5:**
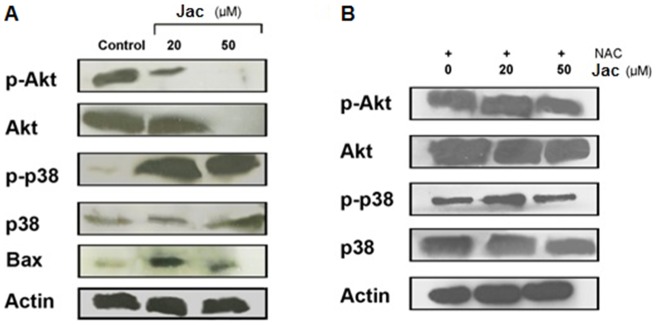
Jacaranone treatment of B16F10-Nex2 cells inhibited Akt pathway activation through ROS generation. Whole-cell extracts from B16F10-Nex2 exposed to 0 (control), 20 or 50 µM jacaranone (Jac) in the presence (A) or absence (B) of NAC were subjected to Western blotting and probed with phospho-p38, phospho-Akt, total Akt, total p38 and Bax antibodies. Protein levels were normalized to the actin level.

Akt is a serine/threonine kinase that stimulates cell proliferation, inhibits apoptosis and has been associated with melanoma progression and prognosis [21]. In this context, Govindarajan et al. demonstrated that melanoma cells overexpressing Akt led to rapidly growing tumors *in vivo* [22]. To determine whether downregulation of Akt is involved in jacaranone-induced apoptosis in B16F10-Nex2, we evaluated the phosphorylation status of Akt using Western blotting analysis. As shown (Fig. 5A), phosphorylated Akt (p-Akt) was detected in lysates of non-treated cells and treatment with jacaranone reduced the level of p-Akt in a dose-dependent manner. Interestingly, the expression of total Akt was abolished in cells treated with 50 µM jacaranone, suggesting protein degradation at this concentration.

The p38 MAPK is preferentially activated by cell stress-inducing signals (e.g. oxidative stress) and leads to apoptosis in response to several cellular injuries [23]. As p38 is partly downregulated due to Akt activation in human cancer [24], [25] we investigated whether p38 activation could contribute to jacaranone-induced apoptosis. High levels of phosphorylated p38 (p-p38) were observed in whole-cell lysates of B16F10-Nex2 exposed to 20 or 50 µM jacaranone, but not in non-treated cells (Fig. 5A).

Because proapoptotic members of Bcl-2 family are implicated in mitochondria-dependent cell death, we examined the effect of jacaranone on enhancing expression of Bax. In line with our results, expression of the proapoptotic Bax protein was consistently upregulated in B16F10-Nex2 after treatment with 20 and 50 µM jacaranone (Fig. 5A).

The role of ROS in the signaling pathways induced by jacaranone was elucidated by pre-incubation of B16F10-Nex2 cells with NAC before treatment with 20 or 50 µM jacaranone. In this experiment, both concentratios were sublethal due to the high number of cells. As shown in Fig. 5B, preincubation with NAC at 4 mM did not result in a decrease of p-Akt protein level, thus restored Akt function in contrast with the cells that were exposed to jacaranone alone. The presence of the antioxidant NAC prevented the induction of active p38 MAPK expression after 24 h of 20 or 50 µM jacaranone treatment. Moreover, alteration in Bax level was not detected upon NAC incubation (data not shown). These results indicated that the modulation of Akt and p38 MAPK cascades, as well as their downstream target Bax, is ROS-dependent and may contribute to regulation of apoptosis in response to jacaranone.

### Jacaranone improves survival of melanoma-bearing mice

**Figure 6:**
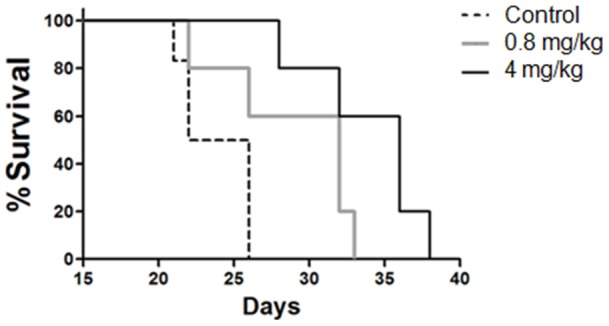
Jacaranone increased survival of B16F10-Nex2 tumor-bearing mice. Animals (5 mice per group) were subcutaneously injected with 5×10^4^ cells and treated with PBS, 0.8 mg/kg or 4 mg/kg of jacaranone on alternate days. Intraperitoneal therapy began one day after inoculation of melanoma cells and was extended for 14 days. Mean survival times were 23.4±2.1 days for untreated tumor control group, 29±4.3 days (*p* = 0.05) for 0.8 mg/kg of jacaranone-treated group, and 34±3.6 days (*p<0.01) for 4 mg/kg jacaranone-treated group.

To determine the effect of jacaranone on tumor development *in vivo*, we used a well-established syngeneic murine melanoma model. B16F10-Nex2 cells were injected subcutaneously into the right flanks of C57BL/6 mice and tumor growth was measured daily. Two treatment groups of 5 mice received 0.8 and 4 mg/kg of jacaranone intraperitoneally every other day for 14 days. A control group received 100 μL of normal saline solution. Mice were removed from the therapy study and sacrificed when tumor size exceeded 3,000 mm^3^. The survival rate (percent value of the number of mice with tumor growth and the total number of mice inoculated in the group) was monitored every day. Treatment of melanoma-bearing mice with 0.8 and 4 mg/kg of jacaranone resulted in a dose-dependent protective effect (Fig. 6). The mean survival times of tumor mice in the 0.8 and 4 mg/kg jacaranone treatment groups were extended to 29±4.3 days (*p* = 0.05) and 34±3.6 days (*p <0.01), respectively, compared with the 23.4±2.1 days of the control group. Moreover, we noted that jacaranone-treated animals maintained healthy physical appearance, normal activity levels and healthy normal-weight throughout the study period. The result shown with 4 mg/kg of jacaranone was confirmed by a second experiment with a group of 6 animals. A positive control with doxorubicin was included which rendered 84% survival of challenged animals (data not shown). Furthermore, no hemolytic activity was detected with jacaranone treatment as described previously [7].

## Discussion

The present work shows that jacaranone is a promising antitumor drug that acts by inducing apoptosis in tumor cells and is especially active against melanoma. Malignant melanoma is the leading cause of skin cancer-related death worldwide [26] and exhibits poor prognosis owing to the lack of efficient treatment. Most chemotherapeutic drugs act through induction of apoptosis [27], and their reduced efficacy in melanoma likely is related to the high resistance of melanoma cells compared to other malignant cell types [28]. Although several therapy strategies have been tested, beneficial effects remain limited, inducing satisfactory responses in a minority of patients [29]. Therefore, novel therapeutic approaches are needed. In this context, plant-derived compounds have played an important role in the development of promising new anti-cancer agents [30].

Jacaranone exhibits potent cytotoxic activity against several cancer cell lines (Table 1) and we demonstrate that this compound induces apoptosis in melanoma cells. The effect of jacaranone was confirmed by detection of surface exposed phosphatidyl serine, DNA fragmentation, loss of mitochondrial Δ*ψ*m, and activation of caspases in B16F10 cells. Also, in agreement with these results, jacaranone treatment rendered tumor cells with nuclear condensed chromatin and nuclear membrane alterations, as well as plasma membrane blebbing. Jacaranone-induced apoptosis involves ROS generation, since concomitant incubation with the antioxidant NAC prevented cell death. We associated production of ROS by jacaranone with the well-known redox cycling capability of quinone-containing substances. During redox cycling, quinoid substances in their reduced states (semiquinones) can yield its extra electron to oxygen with the formation of superoxide anions, beginning a cascade that generates H_2_O_2_ and hydroxyl radicals (.OH) [31], [32]. In addition, we showed that jacaranone induces Akt inactivation, p38 MAPK phosphorylation and expression of proapoptotic protein Bax. Lastly, jacaranone not only showed a remarkable antitumor effect *in vitro* against a number of human cancer cell lines but also acted *in vivo* increasing survival of melanoma-bearing mice in the B16F10-Nex2 syngeneic melanoma model.

As a naturally occurring benzoquinone, jacaranone generates ROS causing an oxidative stress, with enhanced activity of the antioxidant defense system and consequent mitochondrial damage [5], [33]. Interestingly, our data using fluorescent dye TMRE revealed that jacaranone first caused hyperpolarization of the mitochondrial membrane, which is recorded to be one of the earlier key event in apoptosis [34]–[36]. It has been proposed that mitochondrial hyperpolarization may result in a more reduced state of electron carriers responsible for single-electron transfer to oxygen producing primarily the superoxide anion radical, [36] a precursor of most other ROS, also involved in the propagation of oxidative chain reactions [37]. The breakdown of the mitochondrial membrane potential after 2–24 h incubation with jacaranone was detected in melanoma cells.

Mitochondrial permeabilization as well as the leakage of proteins normally confined to mitochondria may increase ROS release and determine the catabolic features of cell death [17]. Besides being predominantly implicated in damaging cellular structures, ROS also play an important role in various physiological aspects of intracellular signaling and regulation. It has been established that ROS interfere with the expression of a number of genes and signal transduction pathways [5].

As shown, the apoptotic effect of jacaranone involves caspase activation. Caspase-9, which was found to be activated at early times in jacaranone-induced cell death process, is an upstream caspase in the mitochondria-dependent pathway [38]. Mitochondrial dysfunction, characterized by loss of mitochondrial transmembrane potential and opening of mitochondrial permeability transition pores, is an effect caused by many apoptotic stimuli either directly or indirectly [39]. In consequence, these organelles release various important proteins to the cytosol to terminate the apoptotic process, including cytochrome c, which is responsible (along with Apaf-1) for the activation of caspase-9.

Caspase-2 activation is another mechanism by which apoptotic stimuli may affect mitochondrial function. It has been shown that caspase-2 engages the mitochondria-dependent apoptotic pathway by inducing the release of cytochrome c and other mitochondrial apoptogenic factors into the cell cytoplasm [40].

Caspase-8 is a major activator of caspase-3 and amplifies the apoptotic signal by directly activating downstream caspases and cleaving BH3 domain-only members of the Bcl-2 family, such as proapoptotic Bid [41]. It has been demonstrated that activation of caspase-8 can occur in death receptor-independent apoptosis [25] and Caspase-2 may act upstream of mitochondria to promote apoptosis [40]. Kim and Hong [42] have reported that Inhibition of caspase-2 suppressed caspase-8 activation by Saikosaponin-a (SSa), but knockdown of caspase-8 did not prevent SSa-induced caspase-2 activation, suggesting that sequential activation of caspase-2 and caspase-8 may occur. It has been clearly demonstrated [43], [44] that ROS triggers activation of caspases during the apoptotic process, including caspase-2 and -8, and facilitates caspase-9 activation via disulfide-mediated interaction with Apaf-1 [45]. Consistent with these previous studies, we detected a significant increase of caspase-2, -8, -9 and -3 activities in B16F10-Nex2 cells after jacaranone treatment.

Akt is a downstream target of the phosphatidylinositol 3-kinase (PI3K) signaling pathway that is activated through phosphorylation of Ser-473/474 and Thr-308/309 [24] and positively regulates cell growth and survival [21]. It has been suggested that Akt is a key regulator of melanoma progression [46]. Indeed, elevated Akt expression in severely dysplastic nevi and melanoma samples, but not in normal and slightly dysplastic nevi of samples from patients with pigmented skin lesions has already been detected, as well as constitutive activation of Akt in several melanoma cell lines [47]. Recent works have reported that oxidative stress can modulate Akt activation [47]–[49]. To elucidate the link between Akt signaling and ROS generation, we investigated the expression of active Akt in B16F10 melanoma cells after treatment with jacaranone. Immunoblotting analysis revealed a high expression of phospho-Akt in non-treated melanoma cells that was downregulated after treatment with jacaranone. It should also be noted that jacaranone treatment at 50 µM reduced the overall expression of Akt. This result raises the possibility that Akt inactivation might also represent a consequence of engagement of the caspase cascade, since Akt degradation by caspases has been reported as a survival signal that allows apoptosis to occur [50].

Another intracellular signaling molecule sensitive to redox modulation is the p38 MAPK, which belongs to the stress-activated protein kinase (SAPK) family [51]. The p38 is an ubiquitous and highly conserved proline-directed serine/threonine kinase considered important in regulation of cell survival, differentiation and apoptosis [52]. The p38 pathway is activated upon cellular stress and often engages signals that suppress proliferation or promote apoptosis, thus it has been referred as a tumor suppressor. Activation of p38 MAPK by ROS has been investigated in a wide variety of experimental models and a number of studies reported that the serine/threonine kinases of the MAPK family can be regulated by oxidants [5]. Particularly in skin cancer, there is evidence that supports a tumor-suppressive role of p38 and most clinically used chemotherapeutics have an anti-proliferative effect on melanoma cells mediated by activation of p38 [53]. In agreement with these observations, we found a marked increase in the expression of p-p38 in B16F10-Nex2 cells after treatment with 20 or 50 µM jacaranone. A crosstalk between the p38 MAPK and PI3K/Akt pathways has been the focus of several studies [54], [55]. Gratton et al. [54] reported that the blockade of Akt signaling stimulates p38-dependent apoptosis, whereas overexpression of constitutively active Akt downregulates p38 activation in endothelial cells. Similarly in our system, ROS generation was responsible for jacaranone-induced p38 MAPK activation and proapoptotic Bax expression. Consistent with the effect on Akt downregulation, pretreatment with NAC blocked jacaranone-induced Akt inhibition compared with cells treated with jacaranone alone. Taken together, our data suggest that ROS mediate the downregulation of Akt and enables the activation of p38 MAPK, thus stimulates apoptosis in jacaranone-treated melanoma cells.

P38-triggered apoptosis is mediated by transcriptional and post-translational mechanisms, which affect death receptors, cell survival pathways, caspases or Bcl-2 family proteins [56]. Besides being well known that Bax is phosphorylated by stress-activated p38 kinase and that phosphorylation of Bax leads to translocation from the cytosol to mitochondria prior to apoptosis [57], there is evidence that p38 may also upregulate Bax expression, thus contributing to apoptosis [58], [59]. Similarly, we found that jacaranone is capable to increase Bax expression in B16F10-Nex2 cells at 20 or 50 µM. Given that Akt can suppress apoptosis through phosphorylation and inactivation of many proapoptotic proteins, such as Bax and Bad [57], [60], Akt downregulation and the increased expression induced by jacaranone may facilitate Bax activation and consequent translocation to the mitochondria, where it forms homo and heterodimers (along with Bak) and creates pores within the outer mitochondrial membrane [17], resulting in release of cytochrome C and activating mitochondria-dependent downstream cascades.

Most importantly, consistent with our findings *in vitro*, jacaranone was able to delay the tumor development in a subcutaneous model with a dose response effect. Based on Akt role as a key regulator of cellular survival and its association with tumorigenesis and melanoma progression, perturbations on Akt signaling pathway may be an important approach to treat this human malignancy. Furthermore, activation of p38 and inactivation of Akt by jacaranone, and its mitochondria-targeted cytotoxicity through ROS may implicate in tumor suppression and sensitization to apoptosis. It should be noted that a smaller effect on ROS generation was observed in normal cells (murine melanocytes and primary cultures of bone marrow macrophages from C57BL/6 mice), suggesting that jacaranone may be employed as a selective cytotoxic agent for the eradication of cancer cells. In conclusion, our results indicate that jacaranone is a promising cancer therapeutic agent potentially able to eliminate tumor cells including those, which are resistant to conventional therapies.
